# Supplementary material for: A Novel Three-Gene Score as a Predictive Biomarker for Pathologically Complete Response after Neoadjuvant Chemotherapy in Triple-Negative Breast Cancer
Source: Cancers (Basel). 2021 May 16;13(10):2401. doi: 10.3390/cancers13102401 (PMC8156144; doi:10.3390/cancers13102401)
Supplement: Supplementary file 1 [file cancers-13-02401-s001.zip › cancers-1221700-supplementary.pdf]

cancers

MDPI

Supplementary Materials

A Novel Three-Gene Score as a Predictive Biomarker for Pathologically Complete Response after Neoadjuvant Chemotherapy in Triple-Negative Breast Cancer

Masanori Oshi, Fernando A. Angarita, Yoshihisa Tokumaru, Li Yan, Ryusei Matsuyama, Itaru Endo and Kazuaki Takabe

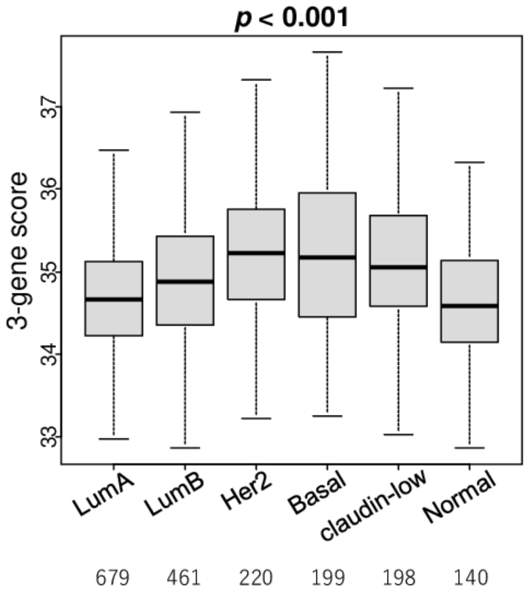

Figure S1. Association of 3-gene with PAM50 subtype in the METABRIC cohort. Boxplots of 3-gene by PAM50 subtype (LumA, LumB, Her2, Basal, claudin-low, and Normal).

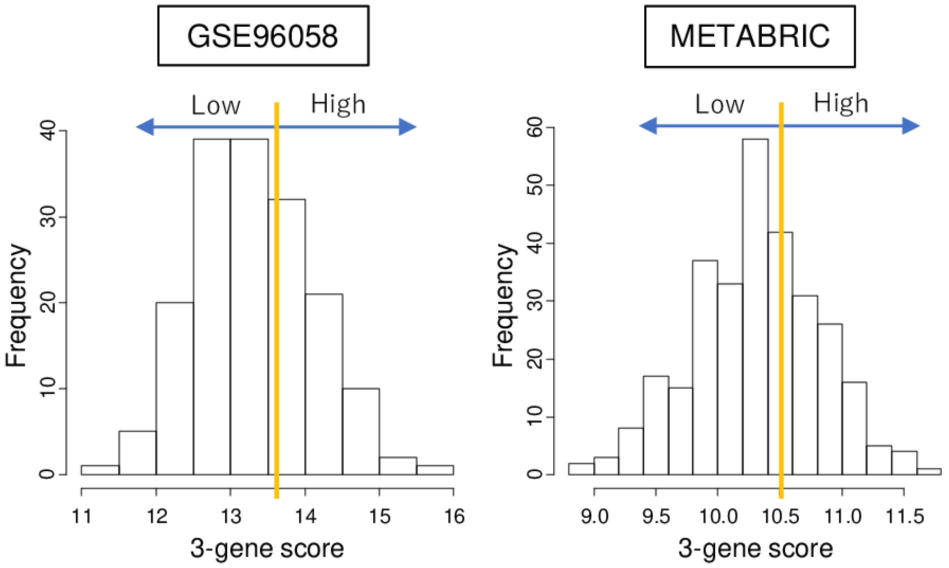

Figure S2. Histogram of the 3-gene score with cut-off line (top one-third) in the GSE96058 and METABRIC cohort.

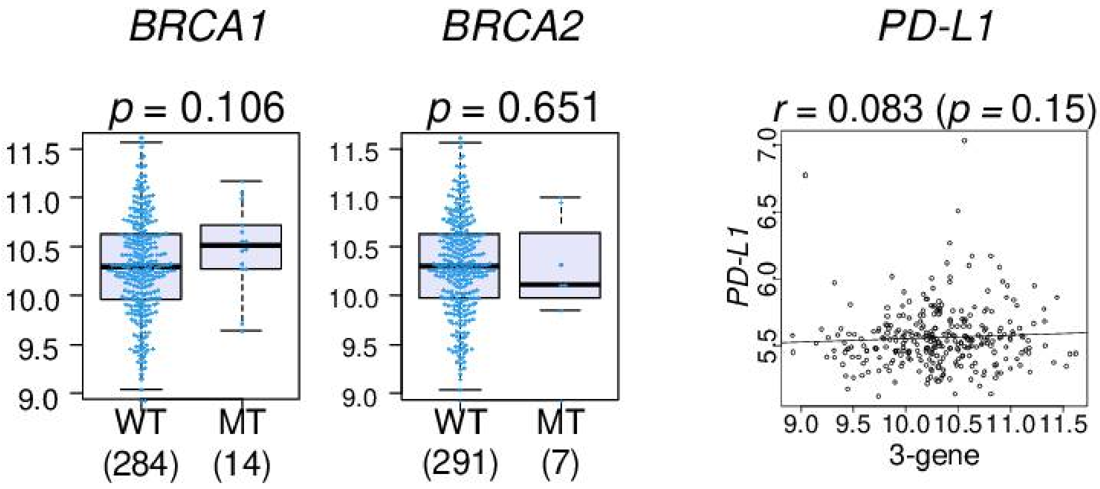

Figure S3. The association of the 3-gene score with *BRCA1* and *BRCA2* mutation, and *PD-L1* expression in TNBC in the METABRIC cohort. Boxplots with Mann-Whitney U *p*-value of the 3-gene score by *BRCA1* and *BRCA2* mutation (MT) and non-mutation (WT) group, and correlation plot with spearman rank correlation value between the 3-gene score and *PD-L1* expression in TNBC.

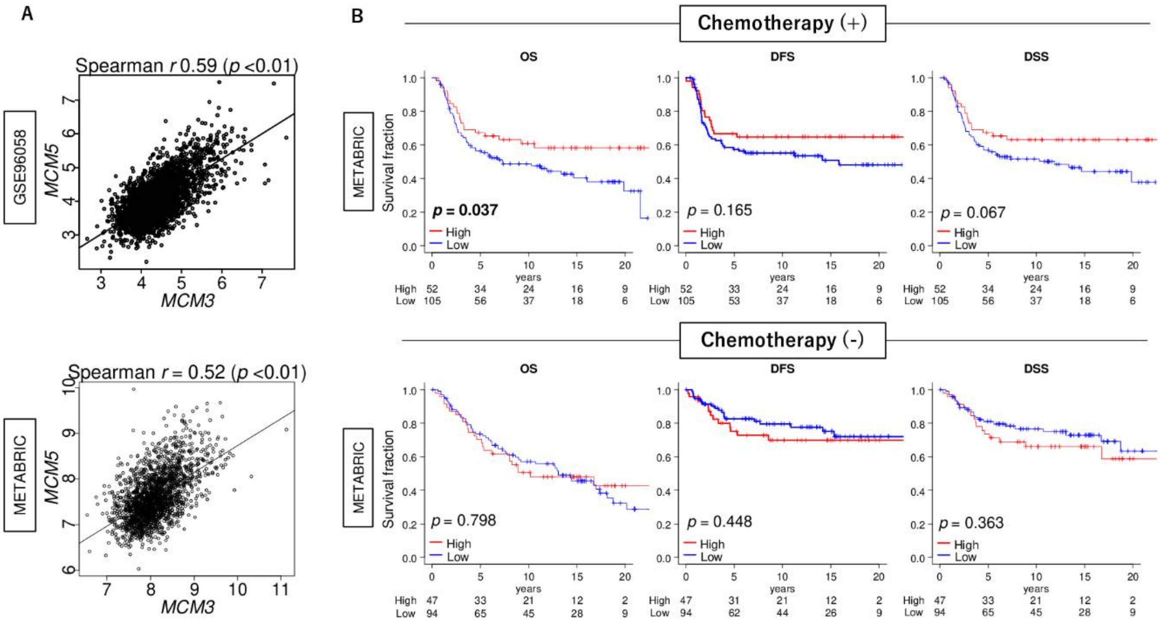

Figure S4. Association between the 3-gene score using *MCM5* and the survival of patients with TNBC with or without chemotherapy. (**A**) Correlation plots between *MCM3* and *MCM5* gene expression in the GSE96058 and METABRIC cohorts. Spearman rank correlation was used to the analysis. (**B**) Kaplan-Meier plots of comparison between low and high 3-gene (using *MCM5*) score groups for Overall Survival (OS), disease-free survival (DFS) and Disease Specific Survival (DSS) in treatment group and non-treatment group in the METABRIC cohort. The top one third was defined as the high score group within cohort. Log rank test was used to calculated the *p* value.

Table S1. Aria under the curve (AUC) of each gene in E2F target gene sets in TNBC in the GSE25066 cohorts.

Gene	*AUC*
CDKN2C	0.687
DEK	0.671
MCM5	0.666
TRA2B	0.659
MCM3	0.656

USP1	0.653
RPA1	0.645
STAG1	0.644
PRIM2	0.643
MSH2	0.642
RFC2	0.641
DEPDC1	0.640
WEE1	0.637
MCM6	0.633
PCNA	0.633
MCM2	0.632
PRDX4	0.628
PLK4	0.626
PAICS	0.623
H2AFZ	0.622
MELK	0.622
NASP	0.621
RBBP7	0.619
RAD51AP1	0.617
CCP110	0.617
RAD51C	0.615
ANP32E	0.615
CHEK1	0.614
BARD1	0.613
NUP153	0.610
ASF1A	0.610
TMPO	0.610
HMGB2	0.610
RAN	0.607
CKS1B	0.606
CDKN1B	0.603
CDKN2A	0.601
RPA3	0.601
KPNA2	0.601
SMC4	0.599
GINS1	0.598
TUBB	0.595
PSIP1	0.595
RAD50	0.595
RACGAP1	0.594
KIF4A	0.594
TOP2A	0.593
CENPM	0.593
RANBP1	0.592
UNG	0.590
DONSON	0.588
EZH2	0.588
KIF18B	0.585
MAD2L1	0.584
CDCA3	0.583
AURKB	0.583
HELLS	0.582
NOLC1	0.580
DNMT1	0.580
TP53	0.579
CBX5	0.578
TIPIN	0.578

GINS3	0.578
RRM2	0.578
PSMC3IP	0.577
ESPL1	0.577
CCNE1	0.574
SMC3	0.574
CENPE	0.574
GINS4	0.573
MLH1	0.573
SNRPB	0.572
BRCA1	0.571
POLD3	0.569
DIAPH3	0.568
CHEK2	0.568
CDK1	0.567
CDCA8	0.567
TUBG1	0.567
SRSF1	0.567
MXD3	0.567
UBE2S	0.566
RFC3	0.566
ATAD2	0.565
ORC2	0.565
CTPS1	0.565
CDC20	0.564
CDC25A	0.564
MKI67	0.564
RFC1	0.564
TK1	0.563
SYNCRIP	0.563
CKS2	0.563
DCK	0.563
CCNB2	0.563
XPO1	0.563
E2F8	0.562
SPAG5	0.562
LIG1	0.561
TBRG4	0.560
PPP1R8	0.560
PPM1D	0.560
ILF3	0.560
POLE	0.559
CDK4	0.557
LMNB1	0.557
PNN	0.557
GSPT1	0.557
BIRC5	0.556
PLK1	0.555
KIF2C	0.554
POLA2	0.554
MCM7	0.553
XRCC6	0.551
NBN	0.551
STMN1	0.551
HMMR	0.550
SHMT1	0.549
MYBL2	0.548

NUDT21	0.546
LUC7L3	0.545
DDX39A	0.545
EXOSC8	0.543
DLGAP5	0.542
CDC25B	0.542
SPC25	0.542
PA2G4	0.539
RAD21	0.538
NUP205	0.537
MYC	0.537
PRKDC	0.536
HMGB3	0.536
TRIP13	0.533
POLD1	0.532
DUT	0.531
HMGA1	0.530
LBR	0.530
CDKN1A	0.528
PTTG1	0.528
AK2	0.527
ING3	0.527
MCM4	0.526
PDS5B	0.525
BUB1B	0.524
CTCF	0.522
EED	0.521
KIF22	0.521
BRCA2	0.521
ZW10	0.520
SSRP1	0.519
DCLRE1B	0.519
SMC1A	0.519
POP7	0.518
NUP107	0.518
NAP1L1	0.518
CDKN3	0.518
NCAPD2	0.517
TIMELESS	0.517
DSCC1	0.516
IPO7	0.516
TFRC	0.516
ORC6	0.515
PAN2	0.515
CSE1L	0.514
MTHFD2	0.513
NME1	0.511
POLD2	0.511
H2AFX	0.511
SPC24	0.511
DCTPP1	0.510
TACC3	0.510
RAD1	0.510
SMC6	0.509
SUV39H1	0.509
HUS1	0.509
RNASEH2A	0.508

EIF2S1	0.508
MRE11A	0.507
SLBP	0.507
ASF1B	0.506
RPA2	0.506
UBR7	0.505
HNRNPD	0.501
PRPS1	0.500

Table S2. Triple-negative breast cancer cell lines.

Cell Line Name
BT20
BT549
CAL120
CAL148
CAL51
CAL851
DU4475
HCC1143
HCC1187
HCC1395
HCC1599
HCC1806
HCC1937
HCC2157
HCC38
HCC70
HDQP1
HMC18
HS578T
MDAMB157
MDAMB231
MDAMB436
MDAMB453
MDAMB468
MFM223
SUM102PT
SUM1315MO2
SUM149PT
SUM159PT
SUM185PE
SUM229PE
